# Supplementary material for: The genome sequence of the Dusky Hook-tip, Drepana curvatula (Borkhausen, 1790) (Lepidoptera: Drepanidae)
Source: Wellcome Open Res. 2026 Sep 14;11:647. [Version 1] doi: 10.12688/wellcomeopenres.27801.1 (PMC13653106; doi:10.12688/wellcomeopenres.27801.1)
Supplement: Peer review history

# Reviewer report: Reviewer response for version 1

**DOI:** 10.21956/wellcomeopenres.30594.r170854

**Published:** 2026-10-09

**Authors:** Vahap Eldem

**Affiliations:** 1 Istanbul University, Istanbul, Turkey

The authors describe a chromosome-scale, haplotype-resolved assembly of the Dusky Hook-tip, *Drepana curvatula*. The assembly is of very high quality, with a contig N50 of 6.53 Mb, an estimated QV of 64.6, 99.96% of the haplotype 1 sequence assigned to 32 chromosomal pseudomolecules including the W and Z sex chromosomes, and 98.5% BUSCO completeness. The methods follow well-established Tree of Life protocols, the data are publicly available, and the Merian element painting adds useful context for comparative studies of lepidopteran chromosome evolution. This genome will be a valuable resource for future work on Drepanidae and Lepidoptera more broadly.

 There are only a few suggestions I can provide. The specimen appears to have been identified on morphology alone. Since the mitochondrial genome has been assembled, could the authors extract a marker gene such as the COI barcode region and compare it against public databases to confirm the identification? BUSCO was run with v5.5.0 and the lepidoptera_odb10 dataset. Could the authors briefly justify this choice, given that the newer lepidoptera_odb12 dataset is now available? If odb10 was retained for consistency with other Project Psyche assemblies, or because the Merian element painting relies on odb10-based assignments, a sentence stating this would be sufficient. Optionally, odb12 results could be reported alongside. No RNA-seq data appear to have been generated for this specimen, as Table 1 lists only PacBio HiFi and Hi-C data. Could the authors clarify what evidence the Ensembl annotation relied on? For example, it may have used RNA-seq from other individuals or public datasets (in which case, please give the number of samples and tissues) or protein homology from related species. Reporting a BUSCO score for the annotated proteome would also help readers assess annotation completeness.

 Overall, these are minor points that can be readily addressed, and I consider the work to be of an acceptable scientific standard.

Are the datasets clearly presented in a usable and accessible format, and the assembly and annotation available in an appropriate subject-specific repository?

Yes

Are sufficient details of the sequencing and extraction, software used, and materials provided to allow replication by others?

Yes

Are the rationale for sequencing the genome and the species significance clearly described?

Yes

Are the protocols appropriate and is the work technically sound?

Yes

Reviewer Expertise:

Genomics, bioinformatics and evolutionary biology.

I confirm that I have read this submission and believe that I have an appropriate level of expertise to confirm that it is of an acceptable scientific standard.

# Reviewer report: Reviewer response for version 1

**Published:** 2026-09-14

**Authors:** Automated Benchmarking

**Affiliations:** 1 Wellcome Sanger Institute, Hinxton, UK

As this article is a Genome Note, Automated Benchmarking has been used to determine whether the reported assembly passes three defined checks established by the Earth BioGenome Project: the raw and assembled data are publicly available, the Earth BioGenome Project three-part metric is met, and the BUSCO score passes the stated threshold. For Drepana curvatula (ilDreCurv1), all three checks passed. Data available: pass. ENA study PRJEB82272, raw-read accessions ERR13946497 and ERR13947525, and assembly accessions GCA_964345945.1 and GCA_964346345.1 are public. Earth BioGenome Project three-part metric: pass. Haplotype 1 is reported as 6.C.Q64, meeting the 6.C.Q40 reference standard. BUSCO: pass. Haplotype 1, which is proposed as the reference assembly for this species, has 98.5% complete BUSCOs (98.3% single-copy and 0.3% duplicated). BUSCO software version: 5.5.0; lineage dataset: lepidoptera_odb10. It meets the target of greater than 90% complete and single-copy BUSCOs. This decision uses values reported in the Genome Note and public accession records.

Reviewer Expertise:

NA

I confirm that I have read this submission and believe that I have an appropriate level of expertise to confirm that it is of an acceptable scientific standard.
